# Contribution of Trabecular Bone Score to Fracture Risk Stratification Beyond Bone Mineral Density in Women with Rheumatoid Arthritis: Results from a Cross-Sectional Cohort

**DOI:** 10.31138/mjr.230725.rac

**Published:** 2026-03-01

**Authors:** Afnane Ismaili, Anas Chbihi, Ilyass Chergaoui, Anas Kherrab, Merieme Ghazi, Imane El Bouchti, Radouane Niamane

**Affiliations:** 1 Rheumatology Department, CHU Mohammed VI, Cadi Ayyad University, Marrakech, Morocco;; 2 Rheumatology Department, Avicenne Military Hospital, Cadi Ayyad University, Marrakech, Morocco

**Keywords:** rheumatoid arthritis, osteoporosis, bone mineral density, trabecular bone score, vertebral fractures

## Abstract

**Objective:**

To assess the contribution of trabecular bone score (TBS) to fracture risk stratification, in relation to bone mineral density (BMD) and vertebral fractures, in women with rheumatoid arthritis (RA).

**Methods:**

This monocentric cross-sectional study included 152 women with RA who underwent dual-energy X-ray absorptiometry (DXA) with TBS evaluation. BMD was measured at the lumbar spine and femoral neck. TBS values were categorised as normal (≥1.300), partially degraded (1.200–1.299), or degraded (≤1.200). Vertebral fractures were identified by vertebral fracture assessment (VFA). Crude and multivariate logistic regression analyses were performed to identify predictors of degraded TBS.

**Results:**

Based on BMD, 38.8% of patients were osteoporotic, while 69.1% had degraded TBS values. Vertebral fractures were detected in 30.3% of patients. Notably, 23.6% showed discordance, with TBS values more degraded than expected from their BMD category. In multivariate analysis, lower lumbar spine BMD (OR 3.62, 95% CI 1.67–7.83, p=0.001) and longer corticosteroid exposure (OR 1.87, 95% CI 1.01–3.45, p=0.046) were independently associated with degraded TBS.

**Conclusion:**

TBS provides complementary information to BMD in evaluating bone quality and fracture risk in RA. Its integration into clinical practice may improve fracture risk stratification in this high-risk population.

## INTRODUCTION

Osteoporosis is a silent, progressive skeletal disorder characterised by reduced bone strength and increased fracture risk, particularly among patients with chronic inflammatory diseases such as rheumatoid arthritis (RA).^1,2^ In RA, bone fragility is multifactorial and driven by chronic systemic inflammation, physical inactivity, and prolonged glucocorticoid use.^3–5^ Osteoporosis has been shown to significantly contribute to adverse outcomes, including increased mortality in RA patients.^6^ While bone mineral density (BMD) assessed by dual-energy X-ray absorptiometry (DXA) remains the standard for diagnosing osteoporosis, it does not fully capture bone microarchitecture or quality.^7^ A substantial proportion of fragility fractures occur in individuals whose BMD values are above the osteoporosis threshold, underscoring the limitations of BMD alone in fracture risk assessment.^8,9^ This discordance is particularly relevant in RA patients, especially those on long-term glucocorticoids, who may present with apparently normal BMD despite compromised bone microstructure.^10^

To address this gap, the trabecular bone score (TBS) was developed as a non-invasive analytical tool derived from lumbar spine DXA images. TBS evaluates pixel grey-level variations to infer bone microarchitecture and has been shown to independently predict fracture risk, even in patients with normal BMD.^11–14^ Several studies, including recent analyses, support its utility in refining fracture risk stratification in high-risk populations, such as RA patients.^15,16^

This study aimed to assess the utility of TBS in evaluating bone fragility and vertebral fracture risk in women with RA, and to explore the potential discordance between BMD and TBS measurements in this population.

## METHODS

### Study Design and Population

This monocentric cross-sectional study was conducted in accordance with the Strengthening the Reporting of Observational Studies in Epidemiology (STROBE) guidelines, with the completed STROBE checklist provided as Appendix 1.

Women aged ≥18 years, diagnosed with rheumatoid arthritis (RA) according to the 2010 American College of Rheumatology/European League Against Rheumatism (ACR/EULAR) classification criteria, and who underwent dual-energy X-ray absorptiometry (DXA) with Trabecular Bone Score (TBS) evaluation between October 2023 and June 2024, were consecutively included. Eligible patients were required to have both lumbar spine and femoral neck Bone Mineral Density (BMD) measurements, with TBS evaluation performed during the same DXA session.

Patients were excluded if they were male, had other metabolic bone diseases such as primary hyperparathyroidism, osteogenesis imperfecta, or Paget’s disease, a history of malignancy or bone metastases, incomplete clinical or imaging data, or pregnancy. Additional exclusion criteria included a body mass index (BMI) above 35.5 kg/m^2^, which invalidates TBS analysis, as well as structural vertebral or femoral abnormalities interfering with DXA or TBS interpretation.

### Data Collection

Clinical and demographic data were extracted from anonymised medical records and verified during outpatient follow-up visits. Collected variables included: Fracture risk factors: age, BMI, menopausal status, comorbidities (diabetes, hyperthyroidism, hyperparathyroidism), personal and family history of fragility fractures, smoking status, calcium intake, and physical activity level;

RA characteristics: disease duration, DAS28 score, rheumatoid factor and ACPA status, presence of structural damage (erosive and/or deforming disease), and functional disability (HAQ score);

Treatment variables: type and duration of glucocorticoid therapy (oral, IV, or intra-articular), cumulative corticosteroid dose (mg), use of conventional synthetic or biologic DMARDs, and osteoporosis treatments. The cumulative corticosteroid dose was calculated by summing all oral, intravenous, and intra-articular administrations documented in the patients’ medical records from RA diagnosis to the date of DXA. In cases of missing data, standard treatment regimens were used for estimation.

Fall risk factors: neurological disorders, visual or auditory impairment, sarcopenia, use of fall-risk medications, and environmental hazards.

Bone and Fracture Assessment

DXA was performed at the lumbar spine (L1–L4) and proximal femur using a Hologic Discovery QDR scanner. BMD results were expressed as g/cm^2^, T-scores, and Z-scores.

Trabecular Bone Score (TBS) was calculated from lumbar spine DXA images using TBS iNsight® software (Medimaps, Switzerland), and categorised as:

Normal: ≥1.300
Partially degraded: 1.200–1.299
Degraded: ≤1.200

Morphometric vertebral fractures were evaluated by Vertebral Fracture Assessment (VFA) and classified according to shape (biconcave, wedge, or crush).

### Statistical Analysis

Data were analysed using SPSS software. Continuous variables were presented as means ± standard deviation or medians (IQR), and categorical variables as frequencies (%). Pearson correlation coefficients (r) were used to assess associations between TBS and continuous variables. Univariate logistic regression analyses were first performed to calculate crude odds ratios (ORs). Subsequently, multivariate logistic regression models were constructed to identify independent predictors of degraded TBS (≤1.200), adjusting for relevant confounders. Statistical significance was set at *p* < 0.05.

No formal sample size calculation was performed, as this study was exploratory and based on all eligible patients available during the study period.

### Ethical Considerations

This study complied with the STROBE guidelines. In accordance with national regulations, retrospective studies based exclusively on anonymised medical records do not require formal ethical approval, and informed consent was waived as all data were collected retrospectively during routine clinical care.

## RESULTS

### Descriptive Analysis

A total of 152 women with rheumatoid arthritis (RA) were included in the study. The mean age was 58.2 ± 9.6 years, and 92.1% of the patients were postmenopausal. The average BMI was 27.4 ± 4.3 kg/m^2^. The median disease duration was 9 years (IQR 4–16), and the mean DAS28 score was 4.4 ± 1.2. Rheumatoid factor (RF) and anti-citrullinated peptide antibodies (ACPA) were positive in 76.3% and 82.9% of cases, respectively. Structural joint damage was reported in 71.7% of patients, and functional disability (assessed by HAQ) was present in 69.7%.

Regarding treatment, 80.9% of patients had received oral corticosteroids, with a median duration of 7 years (IQR 4–12) and a median cumulative dose of 12,000 mg (IQR 6,000–24,000). Most patients were treated with conventional synthetic DMARDs (88.8%), while 16.4% were receiving biologic therapy. Osteoporosis treatment had been initiated in 50.7% of patients. Smoking was reported in 10.5% of the cohort. Morphometric vertebral fractures were detected in 30.3% of patients, and a history of peripheral fragility fractures was noted in 19.1% (**Table 1**).

**Table 1.** History of peripheral fragility fractures.

**Variable**	**Value**
**Age, years**	59.4 ±9.5
**Menopausal status**	93.4%
**BMI, kg/m^2^**	28.2 ±4.4
**RA duration, years**	11.1 ±7.9
**DAS28 score**	4.06 ± 1.23
**RF positive**	73%
**ACPA positive**	65.8%
**Structural damage**	68.4%
**Functional disability (HAQ)**	0.96 ±0.6
**Oral corticosteroids**	77%
**Duration of corticosteroid use, years**	6.7 ±5.4
**Cumulative corticosteroid dose, mg**	16,364.5 ± 14,048.2
**Conventional DMARDs**	97.4%
**Biologic therapy**	6.6%
**Osteoporosis treatment**	53.9%
**Smoking**	10.5%
**Vertebral fractures**	30.3%
**Fragility Fractures**	12.5%

### Bone Microarchitecture and Fracture Risk

**Figure 1.**
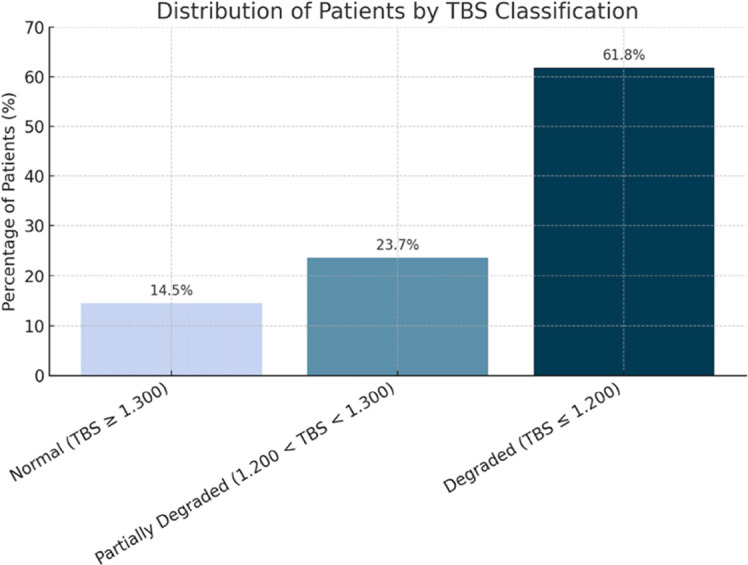
Distribution of patients by TBS classification.

Based on DXA, 38.8% of patients were classified as osteoporotic, 45.4% as osteopenic, and 15.8% had normal BMD. However, TBS analysis revealed a more pronounced impairment of bone quality: 69.1% had degraded TBS (≤ 1.200), 23% had partially degraded TBS (1.200–1.299), and only 7.9% had normal TBS (≥ 1.300) (**Figure 1**).

Vertebral fracture assessment (VFA) detected morphometric vertebral fractures in 30.3% of patients, predominantly of biconcave shape. Notably, 23.6% of patients had TBS values more degraded than expected based on their BMD category, indicating a clinically relevant discordance between bone density and bone quality.

To further explore this discordance, we created a cross-tabulation matrix combining TBS, BMD categories, and vertebral fracture status (**Table 2**).

**Table 2.** Cross-tabulation matrix combining TBS/BMD categories, and vertebral fracture status.

**TBS Category**	**BMD Category**	**Fracture: Yes**	**Fracture: No**
**Normal**	Normal	1	5
**Normal**	Osteopenic	0	6
**Partially degraded**	Osteopenic	5	8
**Partially degraded**	Osteoporotic	6	7
**Degraded**	Osteopenic	8	12
**Degraded**	Osteoporotic	10	12

**Figure 2.**
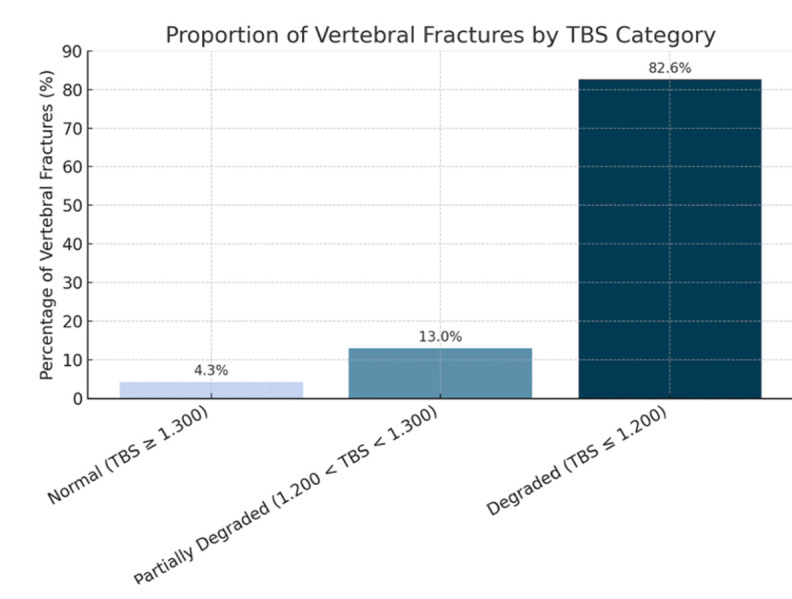
Proportion of vertebral fractures by TBS category.

We then examined the distribution of vertebral fractures according to TBS categories alone (**Figure 2**).

### Correlation Analysis

TBS was positively correlated with lumbar spine BMD (r = 0.482; p < 0.001), T-score (r = 0.424; p < 0.001), and Z-score (r = 0.402; p < 0.001). Moderate correlations were also found with femoral neck BMD (r = 0.354; p < 0.001), T-score (r = 0.320; p < 0.001), and Z-score (r = 0.270; p = 0.001). BMI showed a weak but significant inverse correlation with TBS (r = −0.221; p = 0.006). There was no significant association between TBS and age, disease duration, HAQ score, or cumulative glucocorticoid dose.

### Multivariate Analysis

Multivariate logistic regression was performed to identify independent predictors of degraded TBS (≤1.200). After adjustment for potential confounders, low lumbar spine BMD (OR = 3.62; 95% CI: 1.67–7.83; p = 0.001) and prolonged corticosteroid exposure (OR = 1.87; 95% CI: 1.01–3.45; p = 0.046) remained significantly associated with degraded TBS. Other variables such as BMI and presence of vertebral fractures were not significantly associated (**Table 3**).

**Table 3.** Multivariate logistic regression analysis: predictors of degraded TBS (≤1.200).

**Variable**	**Odds Ratio (OR)**	**95% Confidence Interval (Cl)**	**p-value**
**Lumbar spine BMD (g/cm^2^)**	3.62	1.67–7.83	0.001
**Corticosteroid exposure (years)**	1.87	1.01–3.45	0.046
**BMI (kg/m^2^)**	1.05	0.95–1.17	0.336
**Presence of vertebral fracture**	1.32	0.63–2.78	0.457

## DISCUSSION

This study confirms the added value of the Trabecular Bone Score (TBS) in assessing bone quality among women with rheumatoid arthritis (RA). Beyond bone mineral density (BMD), TBS provided complementary information, with nearly 70% of patients showing degraded trabecular microarchitecture and more than one-fifth presenting worse TBS than expected for their BMD category. This discordance underscores the limitations of relying solely on densitometric measures for fracture risk stratification in RA.^8–10^

Our results align with previous reports showing that a substantial proportion of fragility fractures occur in patients with BMD values above the osteoporosis threshold.^8,9^ TBS, derived from DXA images of the lumbar spine, reflects trabecular microarchitecture and thus captures bone strength parameters not accounted for by BMD.^11–14^ In our cohort, degraded TBS was strongly associated with lower lumbar spine BMD and longer corticosteroid exposure, confirming the negative effect of glucocorticoids on bone quality through impaired bone formation and increased resorption.^2,4,10^ These findings are consistent with experimental and clinical data, and reinforce the importance of integrating TBS into bone health assessment in glucocorticoid-treated RA patients. Recent studies further support this added predictive value, particularly in RA patients receiving long-term glucocorticoids, where TBS has been shown to improve fracture risk discrimination beyond BMD.^17^ From a clinical perspective, the present study provides several relevant insights. More than 30% of patients had morphometric vertebral fractures, the majority occurring in those with degraded TBS, which highlights the clinical utility of TBS in identifying high-risk individuals who may otherwise be overlooked when only BMD is considered. Incorporating TBS into the FRAX® algorithm has been shown to improve fracture prediction and treatment decision-making in RA.^14,18^ Our findings therefore support the combined use of TBS and BMD in daily practice.

Compared with previous studies in European and North American populations, our data extend current knowledge by analysing a North African cohort, which remains underrepresented in the literature. This adds external validity to the role of TBS in RA across diverse clinical settings. Furthermore, the relatively high cumulative glucocorticoid dose observed in our cohort likely reflects local prescribing practices, long disease duration, and the limited availability of biologic therapies during part of the study period. These factors should be considered when interpreting our findings. Similarly, the proportion of patients treated with biologics was lower than expected according to current EULAR recommendations.^19^ This can be explained by barriers to access in our setting, including socioeconomic constraints and health system limitations. Addressing these contextual factors is important when comparing results across different populations.

Another methodological point concerns menopausal status. Although this information was not systematically recorded, the vast majority of our cohort was postmenopausal, and the small minority of premenopausal women (7.9%) is unlikely to have influenced the overall results. Future studies with systematic hormonal assessment would nevertheless provide a more precise evaluation of this factor.

This study has some limitations. Its retrospective design may introduce biases, and the monocentric recruitment could limit generalisability. Furthermore, the absence of inflammatory biomarkers such as C-reactive protein or erythrocyte sedimentation rate prevented adjustment for disease activity, which is a major determinant of bone loss in RA.^3–5,7^ Despite these limitations, the relatively large sample size and systematic assessment of both BMD and TBS strengthen the robustness of our findings.

In summary, this study adds to the growing body of evidence supporting the clinical relevance of TBS in RA. By capturing alterations in bone microarchitecture beyond densitometric parameters, TBS improves fracture risk stratification in a high-risk population and may guide more personalised therapeutic strategies.^13–15,18^ Prospective multicentre studies with longer follow-up are warranted to confirm the predictive value of TBS for incident fractures and to further explore its integration into fracture risk algorithms tailored to RA patients.

## CONCLUSION

In women with rheumatoid arthritis exposed to long-term glucocorticoid therapy, the Trabecular Bone Score (TBS) provided complementary information on bone quality and fracture risk beyond that obtained with bone mineral density (BMD). Nearly one-quarter of patients showed discordance between TBS and BMD, including individuals with non-osteoporotic BMD who nevertheless had degraded trabecular microarchitecture and vertebral fractures.

These findings underline the clinical relevance of incorporating TBS into routine DXA evaluation for RA patients, particularly in those with borderline BMD values or additional risk factors. By improving fracture risk stratification, TBS may help optimise therapeutic decision-making in this high-risk population.

Further prospective, multicentre studies are warranted to confirm the predictive value of TBS for incident fractures and to validate its integration into clinical algorithms and fracture risk assessment tools, such as FRAX, in rheumatoid arthritis.

## Data Availability

The datasets analysed during this study are not publicly available due to patient confidentiality but are available from the corresponding author on reasonable request.

## EDITING AND AI STATEMENT

No professional editing agency was involved in this work. AI-based tools were used only for language editing. All text and content were carefully reviewed, corrected, and validated by the authors, who take full responsibility for the final content.

## References

[1] Compston J McClung MR Leslie WD. Osteoporosis. Lancet 2019;393(10169):364–76. doi:10.1016/S0140-6736(18)32112-3 30696576

[2] Güler-Yüksel M Hoes JN Bultink IEM Lems WF. Glucocorticoids, inflammation and bone. Calcif Tissue Int 2011;89(6):505–16. doi:10.1007/s00223-011-9530-6 29313071

[3] van Staa TP Geusens P Bijlsma JWJ Leufkens HGM Cooper C. Clinical assessment of the long-term risk of fracture in patients with rheumatoid arthritis. Arthritis Rheum 2006;54(10):3104–12. doi:10.1002/art.22117 17009229

[4] Roux C. Osteoporosis in inflammatory joint diseases. Osteoporos Int 2011;22(2):421–33. doi:10.1007/s00198-010-1319-2 20552328

[5] Lekamwasam S Adachi JD Agnusdei D Bilezikian J Boonen S Borgström F A framework for the development of guidelines for the management of glucocorticoid-induced osteoporosis. Osteoporos Int 2012;23(9):2257–76. doi:10.1007/s00198-012-1958-6 22434203

[6] Jadhav PP Patwardhan VG. Effect of hypertension on bone mineral density of patients with rheumatoid arthritis. Mediterr J Rheumatol 2023;34(4):479–85. doi:10.31138/mjr.120923.eoh 38282945 PMC10815520

[7] Kanis JA Melton LJ 3rd Christiansen C Johnston CC Khaltaev N. The diagnosis of osteoporosis. J Bone Miner Res 1994;9(8):1137–41. doi:10.1002/jbmr.5650090802 7976495

[8] Schuit SCE van der Klift M Weel AEAM de Laet CE Burger H Seeman E Fracture incidence and association with bone mineral density in elderly men and women: the Rotterdam Study. Bone 2004;34(1):195–202. doi:10.1016/j.bone.2003.10.001 14751578

[9] Siris ES Chen YT Abbott TA Barrett-Connor E Miller PD Wehren LE Bone mineral density thresholds for pharmacological intervention to prevent fractures. Arch Intern Med 2004;164(10):1108–12. doi:10.1001/archinte.164.10.1108 15159268

[10] Briot K Cortet B Roux C. Glucocorticoids and bone. Joint Bone Spine 2021;88(1):105135. doi:10.1016/j.jbspin.2020.08.00833486108

[11] Silva BC Leslie WD Resch H Lamy O Lesnyak O Binkley N Trabecular bone score: a noninvasive analytical method based upon the DXA image. J Bone Miner Res 2014;29(3):518–30. doi:10.1002/jbmr.2176 24443324

[12] Hans D Goertzen AL Krieg MA Leslie WD. Bone microarchitecture assessed by TBS predicts osteoporotic fractures independent of bone density: the Manitoba study. J Bone Miner Res. 2011;26(11):2762–9. doi:10.1002/jbmr.499 21887701

[13] Leslie WD Aubry-Rozier B Lamy O Hans D. TBS and bone microarchitecture in men and women. Curr Osteoporos Rep 2013;11(2):107–15. doi:10.1007/s11914-008-0017-323467901

[14] Harvey NC Glüer CC Binkley N McCloskey EV Brandi ML Cooper C Trabecular bone score (TBS) as a new complementary approach for osteoporosis evaluation in clinical practice. Bone 2015;78:216–24. doi:10.1016/j.bone.2015.05.016 25988660 PMC4538791

[15] Briot K Paternotte S Kolta S Eastell R Reid DM Felsenberg D Added value of trabecular bone score to bone mineral density for prediction of osteoporotic fractures in postmenopausal women: the OPUS study. Bone 2013;57(1):232–6. doi:10.1016/j.bone.2013.07.004 23948677

[16] Brixen K Kollerup G Charles P Can TBS predict fractures in rheumatoid arthritis? Results from the Danish Osteoporosis Prevention Study (DOOPS). Osteoporos Int 2020;31(2):285–94. doi:10.1007/s00198-019-05169-7

[17] Ruangnopparut R Charoensri S Sribenjalak D Theerakulpisut D Pongchaiyakul C. Trabecular bone score improves fracture risk discrimination in postmenopausal rheumatoid arthritis patients receiving glucocorticoids. Int J Gen Med 2024;17:287–95. doi:10.2147/IJGM.S448659. 38292825 PMC10826709

[18] McCloskey EV Odén A Harvey NC Leslie WD Hans D Johansson H Adjusting fracture probability by trabecular bone score. Calcif Tissue Int 2015;96(6):500–9. doi:10.1007/s00223-015-9980-x 25796374

[19] Smolen JS Landewé RBM Bijlsma JWJ Burmester GR Dougados M Kerschbaumer A EULAR recommendations for the management of rheumatoid arthritis with synthetic and biological disease-modifying antirheumatic drugs: 2022 update. Ann Rheum Dis 2023;82(1):3–18. doi:10.1136/ard-2022-223356 36357155

